# The Development of a Parenteral Pharmaceutical Formulation of a New Class of Compounds of Nitrosourea

**DOI:** 10.3390/ph9040068

**Published:** 2016-11-01

**Authors:** Ludmila Nikolaeva, Natalia Oborotova, Natalia Bunyatyan, Xi Zhang, Ekaterina Sanarova, Anna Lantsova, Olga Orlova, Alevtina Polozkova

**Affiliations:** 1 Ministry of Health of Russian Federation, I.M. Sechenov First Moscow State Medical University, Moscow 119991, Russia; oborotova@mail.ru (N.O.); ndbun@mail.ru (N.B.); zhangxi1007@mail.ru (X.Z.); 2 Ministry of Health of Russian Federation, Federal State Budgetary Scientific Institution, N.N. Blokhin Russian Cancer Research Center, Moscow 115478, Russia; sanarova8686@mail.ru (E.S.); lantsova1979@mail.ru (A.L.); orlova@mail.ru (O.O.); alima9124@rambler.ru (A.P.)

**Keywords:** ormustine, solvents, sterilizing filtration, lyophilization

## Abstract

Despite the rapid development of medical technologies, chemotherapy treatment still occupies an important place in clinical oncology. In this regard, the current research in this area focuses on the synthesis of new highly effective antitumor substances that have minimal side effects and the development of stable pharmaceutical formulations (PF) on their basis. In order to solve this problem, the I. Ya. Postovsky Institute of Organic Synthesis of the Ural Branch of the Russian Academy of Sciences actively sought for original substances, namely, nitrosourea (NU) derivatives, one of the most promising classes of anticancer drugs. As a result of this research, a novel NU derivative was synthesized, namely ormustine, which showed high antitumor activity in preliminary preclinical trials. It is now crucial to develop an ormustine pharmaceutical formulation. Conducted technological studies showed that the most suitable solvent for the drug substance is 0.1 M hydrochloric acid, which ensures its rapid dissolution by ultrasonic treatment. A significant reduction in the concentration of the active ingredient during the storage of the solution required the development of a technique of its lyophilization and the selection of a shaper such as a Kollidon 17 PF. Upon completion of the development of a pharmaceutical formulation of ormustine, its stability after lyophilization was demonstrated, and a sufficient amount of the drug has been acquired for preclinical research.

## 1. Introduction

The incidence of cancer remains common in spite of the discoveries in the field of tumor cells, molecular biology, and a variety of surgical, radiation, and pharmacological intervention methods on various stages and components of tumor growth, which is why it is important to create effective anticancer drugs and improve existing therapies [1]. Therefore, there is now an ongoing search for original drug substances (DS), which is based on carrying out chemical, physical, biological, and pharmaceutical studies [2].

One of the priority directions of research in this area is the development of new anticancer drugs derived from nitrosoureas (NUs) that belong to alkylating agents. A characteristic feature of these compounds that contributes to their biological action is a hydrolytic decomposition in body conditions with a formation of alkylating and carbamoylating particles, and a lack of cross-resistance to typical alkylating agents. The search for new active compounds of NU is associated with attempts to expand the range of the antitumor effect, to reduce side effects and toxicity, and to increase the selectivity of action [3,4,5,6].

The Institute of Organic Synthesis has locally synthesized a new substance fromthe NU class—ormustine. In preliminary biological experiments carried out in the Federal State Budgetary Scientific Institution, the N.N. Blokhin Russian Cancer Research Center, it was found that ormustine induces the death of tumor cells by the mechanism of early apoptosis; moreover, on animals, it exhibits high dose-dependent antitumor activity [7,8,9,10].

Thus, the creation of a stable and effective pharmaceutical composition (PF) of ormustine is a highly relevant area of research.

## 2. Results and Discussion

### 2.1. The Choice of Solvent for the Substance of Ormustine

**Figure 1:**
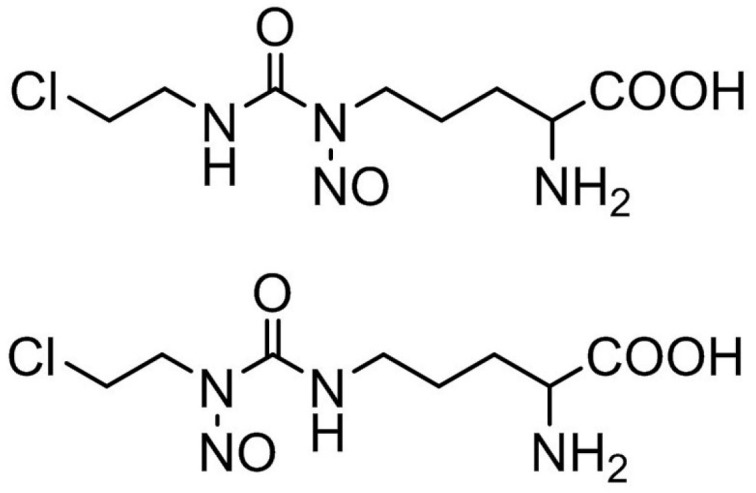
Structural formula of ormustine.

Since the most effective anticancer drugs have an injectable form of PF, ormustine (Figure 1) was also developed for intravenous injection initially as a solution. Since this substance is slowly and sparingly soluble in water, to increase its solubility, different solvents permitted for use in injectable PF were used: 5% solution of mannitol, 2% solutions of Kollidon 17 PF, Kollidon 12 PF and dextran (M_r_~70,000), 10% solution of PEG-1500 and Kollisolv PEG-400, 0.2%, 2%, 4%, and 6% solutions of citric acid; 0.1 M hydrochloric acid (Table 1).

Table 1 shows that a significant increase in the solubility by two times or more was achieved only with the use of citric and hydrochloric acids. However, preliminary studies with the received biological solutions found that the introduction of the citric acid solution at a concentration of 2%–6% to laboratory animals causes death associated with hemolytic reaction [8]. Therefore, we chose to apply a 0.1 M hydrochloric acid solution to increase the solubility of the ormustine substance.

### 2.2. The Choice of Method of Ormustine Dissolution in 0.1 M Hydrochloric Acid

After the final selection of a solvent for ormustine substance, we studied the effect of different solubility intensification techniques—heat to 50 °C, ultrasonic treatment, stirring with magnetic or propeller stirrer—to reduce the time of dissolution and avoid a significant reduction in the concentration of the active substance in solution (Table 2).

To determine the dissolution rate of ormustine in 0.1 M hydrochloric acid, 100 mL of solvent was taken and 2.5 g of the substance was gradually dissolved therein, determining the amount of solute every 10 min until complete dissolution, after which the content of the active substance was evaluated in the solution.

Data presented in Table 2 indicate that the use of ultrasound in the PF preparation process can significantly accelerate the dissolving of the substance and thus maintains the concentration of the active substance practically at baseline. A quicker dissolution of an active ingredient under the influence of the ultrasound is bound to the fact that, under the influence of ultrasonic microstreams, there is an intensive interfusion of liquid layers at the surface. The rate of dissolution of ormustine under the influence of the ultrasound is also increased as a result of cavitational erosion and the adding of solid particles. This considerably increases the interface between the dissolvent and the dissolved substance. While intensification of the substance dissolving using a magnetic propeller stirrer and heating only slightly accelerates the dissolution process, it reduces the concentration of the active substance by more than 10% of the original. Therefore, we can conclude the feasibility of the use of ultrasound technology for ormustine PF.

### 2.3. Choice of Shaper for Lyophilization of Ormustine Solution

A 2.5% solution of ormustine is unstable during storage; within 24 h, there is a decrease in the concentration of the active substance by 3%–6%; therefore, to stabilize and increase the shelf life of PF, it is necessary to conduct lyophilization. Therefore, we examined the effect ofthe inclusion of a shaper in the ormustine solution during freeze drying to form a porous lyophilic mass and provide an absence of significant reduction in the active substance content in the solution during process operations (at least 3 h). During the production of the model compounds, the ormustine substance was dissolved in 0.1 M hydrochloric acid under ultrasound action. After complete dissolution of the substance, one or more auxiliary substances were added to DS: Kollidon 12 PF and 17 PF, lactose, mannitol, and citric acid at various concentrations (Table 3). Then, it was filtered through a nylon filter membrane.

The results presented in Table 3 indicate that the addition of 6% Kollidon 17 PF as a shaper and cosolvent in the model solution compared with the 2.5% ormustine solution without auxiliaries significantly reduced the loss of DS from 7.3% to 1.4% during the preparation of the solution for lyophilization. The solution containing a mixture of 6% Kollidon 12 PF and 0.1% citric acid showed a slight decrease of ormustine losses, while only the addition of 10% Kollidon 12 PF or a mixture of 4% Kollidon 12 PF and 2% lactose destabilized, on the contrary, the active substance by 1.5 times. The addition of 4% lactose and 4% mannitol led to an increased degradation of DS by about 2.5 times. In connection to the above, Kollidon 17 PF at a concentration of 6% was chosen as the most optimal ormustine shaper stabilizing solution.

### 2.4. Development of Lyophilization Mode for the Solution of Ormustine

**Figure 2:**
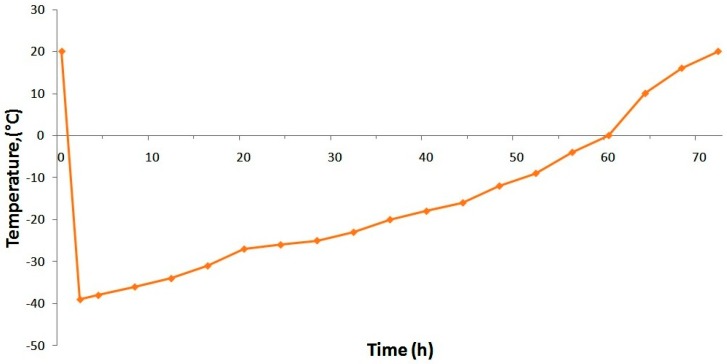
Temperature curve for formulation during drying.

To stabilize ormustine the PF solution, some trial lyophilizations were conducted under various conditions. We evaluated the effectiveness of the proposed mode on the parameters: lyophilization duration and the quality of the obtained lyophilizate (appearance, pH change, loss of DS during lyophilization, and humidity). Being a regime allowing obtainment of the lyophilizate with a good appearance, with little loss of the active ingredient in the shortest time, we selected a mode in which the formulation was frozen at −45 °C on a shelf of a freeze drying unit (a shelf temperature of −45 °C) and maintained this temperature for 3 h; then, the vacuum was switched on. After stabilization of the vacuum, the condenser, and the formulation temperature, heating was performed at the rate of +5 °C/h up to a shelf temperature of −35 °C. Further temperature increase (after passage of the formulation eutectic zone that is −23 °C) was at +3 °C per hour. Upon reaching 0 °C on the formulation, we performed a gradual rise in temperature to +20 °C at +2 °C/h; after reaching a predetermined temperature for the preparation, a final drying (removal of residual moisture) was carried out over the course of 3 h (Figure 2).

### 2.5. The Choice of Solvent for Rehydrating of Lyophilized Ormustine PF Prior to Administration

At the final stage of the technological research, it was necessary to choose a solvent for the rehydrating of the freeze dried product. For this purpose, we evaluated the effect of solutions most commonly used for injecting (0.9% NaCl solution, 5% glucose solution, Ringer’s solution, Hemodes-N, and phosphate buffered saline with a pH of 6.8–7.1) on pH and the physical stability of the solution obtained after rehydration of the ormustine lyophilizate vial (Table 4).

**Figure 3:**
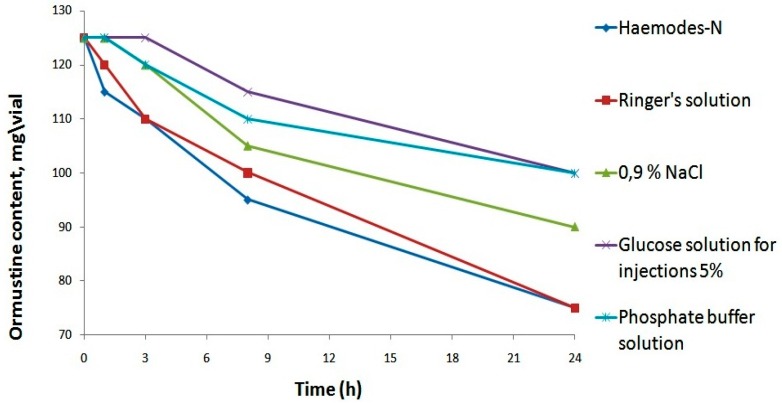
Stability studyof ormustine in PF after rehydration in various solvents.

All solvents provided a true solution, but a significant increase in pH was ensured only through the application of a phosphate buffer solution. Further, in order to select the most appropriate solvent from the five proposed options, we assessed their impact on the stability of the resulting ormustine in the solution after rehydration. For this purpose, ormustine lyophilizate was dissolved in 5 mL of a 0.9% NaCl solution, a 5% glucose solution, a Ringer’s solution, Hemodes-N, and 10 mL of a phosphate buffer solution, and the concentration of active compound was measured at control time intervals—1 h, 3 h, 8 h, and 24 h. (Figure 3).

When Ringer’s and Hemodez-N solutions were used as solvents, 1 h after rehydration of lyophilizate, we observed a reduction in the concentration of the active substance; losses were 4% and 8%, respectively. After 1 day, the ormustine concentration in the solution with the above-mentioned solvents fell to 40% of the original. The use of the isotonic sodium chloride solution and the phosphate buffer solution as solvents allowed us to keep the original concentration of ormustine for 1 h and of the 5% glucose solution for 3 h. The lowest losses of the active substance at all stages of the experiment were observed with the use of the 5% glucose solution and the phosphate buffer solution as solvents. Therefore, further studies for the rehydrating of freeze dried product could use a 5% glucose solution, a phosphate buffer solution, and a 0.9% solution of NaCl.

### 2.6. Results of Preliminary Preclinical Trials

From the results of several preliminary preclinical trials, it was established that, by single intravenous medication administration, ormustine in a 125 mg/kg dose led to the successful treatment of mice with leukoses in a large percentage of cases. In regard to P-388 lymphocytic leukemia, ormustine led to the treatment of mice in 50% of cases; in regard to L-1210 lymphoid leukemia, it was 66.7%; in regard to the cervical cancer (CC), ormustine led to the treatment of all mice in the experimental group. At the same time, extraction for carmustine for strain data was 20% on P-388, 50%–100% on L-1210, and 75% on CC. Ormustine also shows a high therapeutic effect on the B-16 melanoma and the soft Lewis epidermoid carcinoma LLC. The tumor growth inhibition (TGI) on the B-16 melanoma achieves 99.3%–91% within 15 days (the increase in lifespan is 84%) and 99.9%—87% within 14 days on LLC (the increase in lifespan is 84%). On carmustine, TGI by the B-16 melanoma comes up to 89% and a 25%–60% by LLC.

When comparing data on the specific activity of ormustine with other NU derivatives, it becomes obvious that this drug possesses a stronger antitumoral activity on a series of tumors.

A cytotoxicity research was conducted on the Mel Z cellular line of disseminate melanoma of a human subject. The results of the research are given in Table 5.

As the table shows, ormustine possesses a more expressed cytotoxic action in comparison with other drugs.

## 3. Materials and Methods

### 3.1. Preparations and Reagents

The following substances were obtained for this study: ormustine substation (I. Ya. Postovsky Institute of Organic Synthesis of the Ural Branch of the Russian Academy of Sciences, Russia), mannitol (Chimmed, Russia), Kollidon 12 PF, Kollidon 17 PF, Kollisolv PEG-400 (BASF The Chemical Company, Germany), dextran M_r_~70,000 (Sigma-Aldrich GmbH, Germany), PEG-1500 (Chimmed, Russia), 99% sorbic acid (Fluka, Germany), 99% glutamic acid (Merck, Germany), ascorbic acid, reagent grade (Chimmed, Russia), citric acid anhydrous, reagent grade (Ctrobel, Russia), potassium phosphate monobasic, reagent grade (Chimmed, Russia), sodium phosphate dibasic 12-water, reagent grade (Chimmed, Russia), hydrochloric acid, reagent grade (CJSC Mosreaktiv, Russia), 5% glucose solution for infusion (Biosynthesis, Russia), Hemodesum-N solution for infusion (Biok, Russia), Ringer’s solution for infusion (CJSC “Rester”, Russia), and 0.9 % sodium chloride solution for infusion (B. Braun Melsungen AG, Germany).

### 3.2. Equipment

The following equipment was used for this study: a Scales Sartorius LA 1200 S (Sartorius AG, Germany); analytical scales Ohaus Analytical Plus 119 (Ohaus, USA), an ultrasonic bath Transsonic (Elma, Germany), a mechanical overhead Stirrer RZR 2021 Heidolph with a propeller stirring element PR 30 Heidolph (Heidolph, Germany), a glass filter holder Millipore (Millipore, France) with nylon PALL N66 membrane filters with a diameter of 47 mm and a pore size of 0.22 microns (Pall Corporation, USA, LLC Pall Eurasia, Russia), a freeze drying apparatus Minifast DO.2 (Edwards, UK), a spectrophotometer Cary 100 (Varian, Inc., Australia), a pH-meter HANNA pH 211 (Hanna Instruments, Germany), and a Dispensette dispenser (BRAND, Germany).

### 3.3. Study of the Solubility of Ormustine Substance

The study of the solubility of the ormustine substance was conducted visually at 20 ± 2 °C using a variety of solvents and expressed as a percentage by weight/volume [11].

### 3.4. Sterilizing Filtration of Ormustine Solution

Sterilizing filtration was carried out under vacuum using a nylon (filtration using glass filter holder Millipore) and polyethersulfone (included on Stericup vacuum filtration system) membrane filter with a pore diameter of 0.22 microns.

### 3.5. Freeze Drying of Ormustine Solution

Freeze drying of DS was carried out on a freeze-drying apparatus Minifast DO.2. Thus, ormustine solution was filtered immediately after preparation and dosed with 5 mL ormustine solution (empirically established optimum filling volume) in vials of 20 mL, loaded onto the freeze drying shelves, and lyophilized using three different temperature conditions:

1. ormustine solution loading in the bottle on warm shelves with the rapid freezing and uniform temperature rise;
2. ormustine solution loading in the bottle on warm shelves with the slow freezing and uniform temperature rise;
3. ormustine solution loading in the bottle on cold shelves with the rapid freezing and uniform temperature rise.

### 3.6. Potentiometric Determination of pH of Solution and Lyophilizate (upon Rehydration) of Ormustine

The determination of pH of the solutions and the lyophilizate (upon rehydration) of ormustine was carried out potentiometrically.

### 3.7. Quantification of Ormustine in Solution and Lyophilizate

The content of DS in the solutions and lyophilizate was determined spectrophotometrically using a working standard sample substance of ormustine at λ = 396 ± 2 nm.

Dilution of the fresh ormustine solution was performed as follows:

The separation of insoluble ormustineis was carried out by filtering of the solution via sterile nylon membranous filters. Five milliliters of the filtered ormustine solution was transferred with a measuring pipette to a measuring flask with a capacity of 50 mL, make up the volume of the flask with 0.01 M hydrochloric acidum, and mixed.

Lyophilizate dilution was performed as follows:

The contents of the flask were dissolved with 0.01 M hydrochloric acid and quantitatively transferred to a measuring flask with a capacity of 50 mL. The solution volume was brought to a tag by the same dissolvent and mixed. The time between the beginning of the preparation of the test solution and the measurement of its optical density was not to exceed 30 min.

Preparation of the standard solution was performed as follows:

About 125 mg (precise test portion) of ormustine substance was dissolved with 0.01 M hydrochloric acid and quantitatively transferred to a measuring flask with a capacity of 50 mL. The solution volume was brought to a tag by the same dissolvent and mixed. The time between the beginning of the preparation of the test solution and the measurement of its optical density was not to exceed 30 min.

The optical density of the test solution and the standard solution was measured on the spectrophotometer in the absorption maximum at a wavelength of 396 ± 2 nm in the cuvette with a layer thickness of 10 mm, with 0.01 M hydrochloric acid used as a comparison solution.

The ormustine contents X (mg) in the flask were calculated with the following formula:

X = (D_1_ × V_1_ × a_0_)/(D_0_× V_0_), (1)

where D_1_ = the optical density of the test solution; D_0_ = the optical density of the standard solution; V_1_ = the size of dilution of the test solution; V_0_ = the size of dilution of the standard solution; a_0_ = a precise test portion of a standard sample, in mg.

## 4. Conclusions

In the course of this research, aimed at developing a novel injectable NU ormustine PF, a solvent was selected, namely a 0.1 M solution of hydrochloric acid, which was found to significantly increase the solubility of the substance, and the use of ultrasound in the technological process of obtaining the ormustine solution was demonstrated. In order to produce a PF stable in time, a lyophilization regime was designed. For effective application of this procedure after a series of experiments, Kollidon 17 PF was chosen with a concentration of 6% as a shaper, which allowed obtainment of a high-quality lyophilizate. At the final stage, we estimated the effects of solvents for the rehydrating of lyophilized ormustine DS on its stability; as a result, we found that the solvents that ensure the most long-term stability of the resulting DS solution and that do not cause acute toxicity are a 5% glucose solution, a phosphate buffer solution, and a 0.9% NaCl solution. As a result of complex technological research, we developed stable lyophilized ormustine DS, which was then transferred to preclinical studies. The received results of the preliminary preclinical trials provide grounds for the continuation of the research of ormustine, for the study of its cross-resistance with other NU derivatives, and, via combination therapy of tumors, for the rise in efficiency of the treatment of oncologic patients.

## Figures and Tables

**Table 1:** Solubility of ormustine substance in water and solutions of solubilizers.

Solvent	pH	Ormustine Concentration in Solution, %
water for injection	3.9	1.0
5% solution of mannitol	3.5	1.7
2% solution of Kollidon 17 PF	3.5	1.8
2% solution of dextran	3.6	1.7
5% solution of mannitol 2% solution of Kollidon 17 PF	3.6	1.6
0.2% solution of citric acid	2.9	1.5
2% solution of citric acid	2.4	2.0
4% solution of citric acid	2.3	2.5
6% solution of citric acid	2.2	2.5
10% solution of Kollidon 17 PF 0.1% solution of citric acid	3.1	1.6
10 % solution of Kollidon 12 PF 0.1% solution of citric acid	3.2	1.7
10% solution of Kollisolv PEG-400 0.1% solution of citric acid	3.1	1.8
10% solution of PEG-1500 0.1% solution of citric acid	3.2	1.8
0.1 M solution of hydrochloric acid	2.0	2.5

**Table 2:** Effect of different methods on the rate of dissolution of the ormustine substance.

Parameter	Dissolution Method			
	Heating	Ultrasound Treatment	Magnetic Stirrer	Propeller Stirrer
median dissolution rate, g/min	0.7 ± 0.1	0.89 ± 0.05	0.16 ± 0.05	0.35 ± 0.1
ormustine content in the solution after complete dissolution, % of the theoretical content	75 ± 5	99 ± 2	85 ± 3	89 ± 3

**Table 3:** The impact of shapers on the stability of ormustine.

Shaper	Dissolution Time, min	pH	DS Loss within 3 h, %
-	12	2.0	7.3
Kollidon 17 PF 6%	17	2.1	1.4
Kollidon 12 PF 10%	25	2.1	11.5
Kollidon 12 PF 6% citric acid 0.1%	25	2.1	6.4
Kollidon 12 PF 4% lactose 2%	15	2.0	9.8
lactose 4%	16	2.0	19.6
mannitol 4%	22	2.0	16.1

**Table 4:** Changing of pH by adding various amounts of solvents to lyophilized ormustine PF.

Volume of Added Solvent, mL	pH				
	0.9% NaCl	5% Glucose Solution	Ringer’s Solution	Hemodes-N	Phosphate Buffered Saline
5	2.0	2.0	2.0	2.3	not dissolved
10	2.0	2.1	2.1	2.5	6.5
15	2.1	2.2	2.3	2.9	-

**Table 5:** Studying influence of nitrosoureas (NUs) on the Mel Z line cells.

Drug	IC_50_ (mg/mL)		
	24 h	48 h	72 h
Aranoza	0.9	0.9	0.225
Lizomustine	0.125	0.125	0.062
Ormustine	0.125	0.062	0.062

## Abbreviations

The following abbreviations are used in this manuscript:

PF: pharmaceutical formulation
NU: nitrosourea
DS: drug substance
CC: cervical cancer
TGI: tumor growth inhibition
